# Pyrazole Carbohydrazide Derivatives of Pharmaceutical Interest

**DOI:** 10.3390/ph5030317

**Published:** 2012-03-16

**Authors:** Luiza Rosaria Sousa Dias, Raquel Rocha Silva Salvador

**Affiliations:** Laboratório de Química Medicinal (LQMed), Faculdade de Farmácia, Universidade Federal Fluminense (UFF), Rua Mário Viana, 523, Santa Rosa, Niterói 24241-000, RJ, Brazil

**Keywords:** pyrazole, carbohydrazide, biological activity

## Abstract

The main purpose of this paper is to provide an insight into the biological activities of pyrazole derivatives which contain the carbohydrazide moiety.

## 1. Introduction

Clinically biological activity is the result of a chemical compound’s interaction with a human organism. The biological activity is dependent upon the compound’s structure and its physical-chemical characteristics, as well as the biological entity and its mode of therapeutic treatment. Nonetheless, many times chemical compounds reveal a spectrum of different types of biological activity. Some of them are useful in the treatment of definite diseases, where others may cause various side and toxic effects. This complex of activities caused by the chemical compound in biological entities is called the “biological activity spectrum of the substance”.

Hydrazides, carbohydrazides and similar compounds are well known as useful building blocks for the synthesis of a variety of heterocyclic rings. A large number of heterocyclic carbohydrazides and their derivatives are reported to exhibit significant biological activities [1,2] and the carbohydrazide function represents an important pharmacophoric group in several classes of therapeutically useful substances [1,3,4,5,6,7,8]. In this paper, the authors will discuss the biological activity spectrum of various pyrazole derivatives containing carbohydrazide moieties.

## 2. Pyrazole Compounds with Carbohydrazide Moiety Internalized

The core pyrazole structure in general has attracted widespread attention because of the diversity of biological activity shown by derivatives of this nucleus. In fact, one of the first synthetic organic compounds to find use as an important drug was a pyrazolone. Antipyrine (**1**), first prepared in 1887, was used as antipyretic, analgesic and anti-inflammatory. Modifications in the pyrazolone nucleus led to the discovery of dipyrone (**2**), an analgesic and antipyretic drug, still used in several countries in Central and South America as well as in Europe, Asia, and Africa.

**Figure 1:**
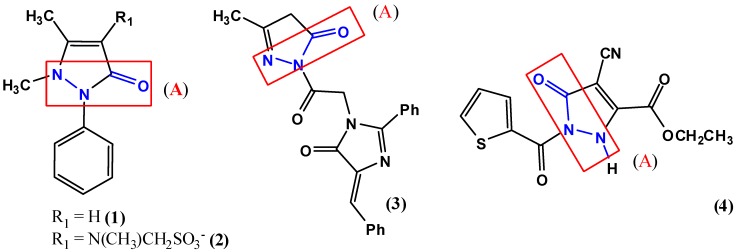
Some pyrazolone derivatives that present biological activities. Antipyretic and analgesic activities could be observed for the derivatives **1** and **2**, anti-inflammatory activity could be observed for the derivatives **1** and **3** and the derivative **4** presents antidepressant activity. In the core pyrazolones we can observe the internalized carbohydrazide functions (**A**).

Pyrazolone derivatives are still under investigation [9,10]. In this way, there are some derivatives with a broad spectrum of activities, including antidepressant and anti-inflammatory effects. 2-{2-[4-(4-Fluorobenzylidene)-2-(4-fluorophenyl)-5-oxo-4,5-dihydroimidazol-1-yl)acetyl}-3-methyl-2,4-dihydro-1*H*-pyrazole-5-one (**3**) is an effective anti-inflammatory agent in the carrageenan induced rat paw edema test [10] and ethyl 1-(thiophene-2-carbonyl)-4-cyano-5-oxo-2,5-dihydro-1*H*-pyrazole-3-carboxylate (**4**) showed good antidepressant activity [9]. Interesting, the carbohydrazide moiety can be observed internalized into the heterocyclic nucleus at the core structure of pyrazolones (Figure 1).

**Figure 2:**
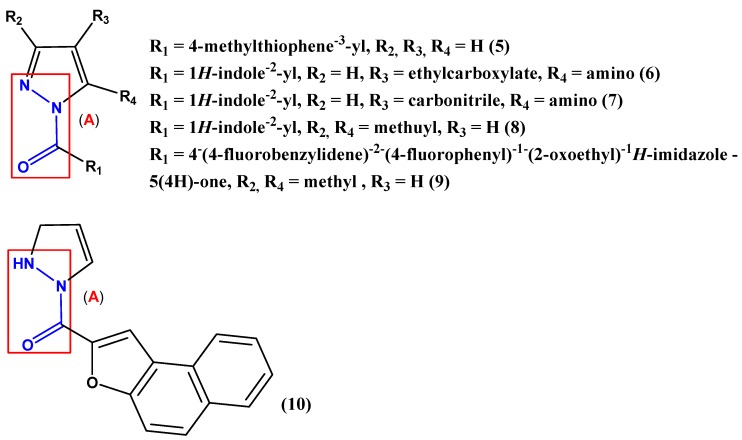
1-*H*-pyrazole derivatives **5**–**9** and 2,3-dihydro-1-*H*-pyrazole derivative **10** that exhibit biological activities. An internalized carbohydrazide function (**A**) can be observed.

In this way some 1-*H*-pyrazole and 2,3-dihydro-1-*H*-pyrazole derivatives that have the internalized carbohydrazide moiety also exhibit interesting biological activities (Figure 2). Antimicrobial activity could be observed for the 1*H*-indole-2yl group (compounds **6**–**8**) with moieties such as 4-methylthiophene-3-yl (compound **5**) and 1-(naphtho[2,1-*b*]furan-2-yl) (compound **10**) connected to the carbonyl group of the carbohydrazide function [6,11,12]. The derivative **5** also exhibited analgesic activity [11]. Only the derivative **9** presented anti-inflammatory activity in the carrageenan induced rat paw edema assay [10].

## 3. Biological Activities of Carbohydrazides Derived from Pyrazole

**Figure 3:**
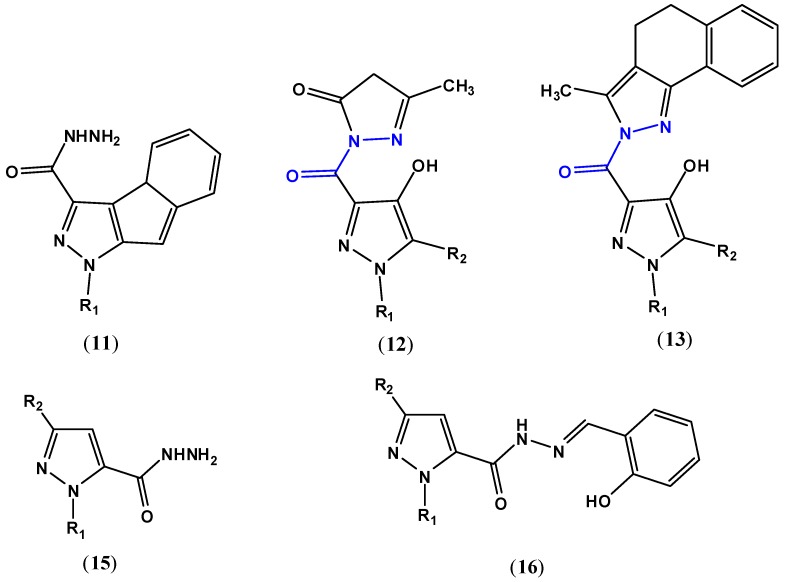
Derivatives that exhibit antitumor activity: 1-*H*-pyrazole-3-carbohydrazide derivatives (**11**, R_1_ = phenyl), 4-hydroxypyrazole derivatives (**12** and **13**, R_1_ = 4-chlorophenyl, R_2_ = H), 1-*H*-pyrazole-5-carbohydrazide derivatives (**15**, R_1_ = 4-*substituted*-benzyl or -C(O)CH_2_-O-phenyl, R_2_ = 4-*substituted*-benzyl or 4-*substituted*-phenyl) and (**16**, R_1_, R_2_ = benzyl or 4-*substituted*-benzyl). The compounds **12** and **13** have the carbohydrazide function (**A**) internalized into a heterocyclic nucleus.

An investigation of the relationship between the pyrazole nucleus and the position of carbohydrazide moiety substituent with biological activity was one of the goals of this study. Substitution with carbohydrazide moiety at position C-3 of the pyrazole ring (Figure 3) seems to provide derivatives that exhibit biological activities such as antitumor (compounds **11**–**13**) and cannabinoid antagonist (compound **14**) [13,14,15,16].

**Figure 4:**
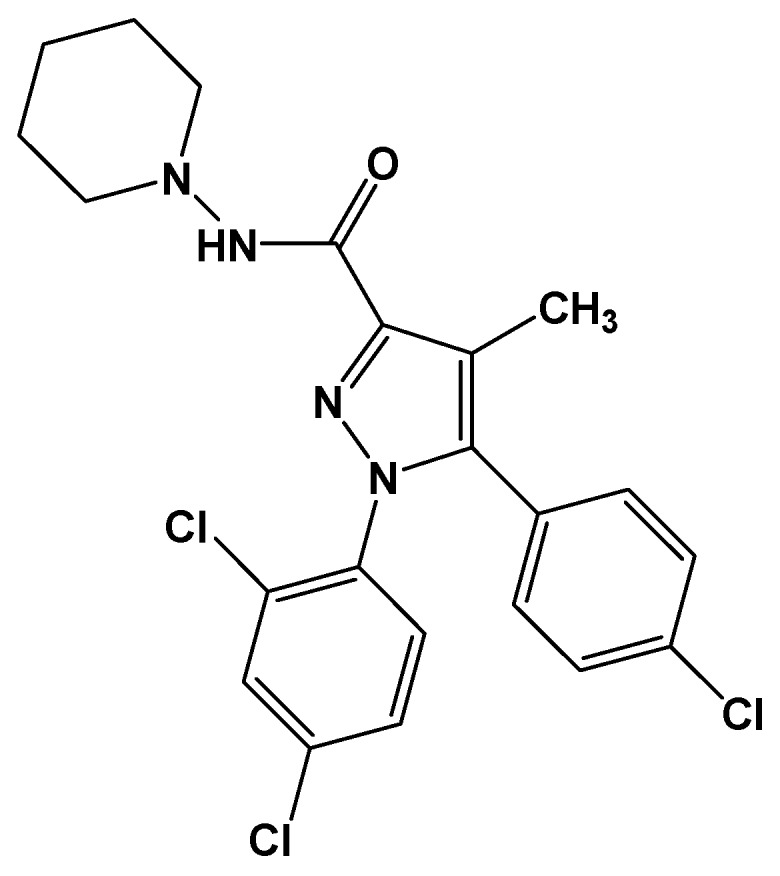
Rimonabant (SR141716, **14**).

*N*-(Piperidinyl)-5-(4-chlorophenyl)-1-(2,4-dichlorophenyl)-4-methyl-1*H*-pyrazole-3-carbohydrazide (**14**, rimonabant, SR141716, Figure 4) is a potent CB1 cannabinoid receptor antagonist. This activity can be investigated to provide a chemical tool for further characterization of the cannabinoid pharmacophore and its relationship to the binding domain of cannabinoid antagonists [15,16]. Its main effect is reduction in appetite [17,18]. 

The anticancer activity of the 4-hydroxypyrazole compounds **12** and **13** was revealed by the broad spectrum of antitumor potential against tumor cell lines at the GI50 and TGI levels, together with a mild cytotoxic activity [14]. The 1-phenylindeno[1,2-*c*]pyrazole **11** also showed potent cytotoxic activity [13].

Substitution with a carbohydrazide moiety at position C-5 of the pyrazole ring seems to provide derivatives that exhibit antitumor activity too (compounds **15** and **16**, Figure 3), beyond fungicidal and herbicidal activities [8,19,20,21,22,23,24].

The research for novel targeted therapies that can induce death in cancer cells or sensitize them to cytotoxic agents involves some 1*H*-pyrazole-5-carbohydrazide derivatives that can inhibit the growth of A549 cells in dosage-dependent and time-dependent manners [19,20]. Salicylaldehyde-pyrazole-carbohydrazide derivatives (compound **16**, Figure 3) have been investigated in inhibition of the proliferation of A549 lung cancer cells [8,21,22,25,26]. In light of the increased anticancer activities of some copper complexes, salicylaldehyde-pyrazole-carbohydrazide derivatives shown to be potent growth inhibitors to A549 cells via inducing apoptosis [22]. 

On the other hand, substitution with a carbohydrazide moiety at position C-4 of the pyrazole ring seems to provide derivatives that exhibit different biological activities such as antinociceptive, antibacterial and antiparasital activities.

The 4-dimethylaminephenyl derivative of 1*H*-pyrazole-4-carbohydrazide, (**17**, ED_50_ 1.41 mg/Kg) showed an antinociceptive activity eleven times more potent than dipyrone (ED_50_ 15.8 mg/Kg) [27,28]. However, the 4-hydroxyphenyl derivative **18** showed potential antimalarial activity targeting the inhibition of *Plasmodium falciparum* cysteine protease [29].

Some 1*H*-pyrazole-4-carbohydrazide derivatives have provided new perspectives on the development of drugs with activities against microbial diseases. Apparently, the carbohydrazide moiety and the nature of the substituents introduced in the *N*-phenyl ring (R_1_ and R_2_) are crucial to determine the potentiality of the antimicrobial activity profile. A series of 1-(4-*substituted*-phenyl)-1*H*-pyrazole-4-carboxylic acid (4-*substituted*-benzilidene)-hydrazide derivatives (**19**) marked leishmanicidal activity on *L. amazonensis* [30,31]. Another series of 1*H*-pyrazole-4-carbohydrazides **20** presented moderate bactericidal and bacteriostatic properties [32].

**Figure 5:**
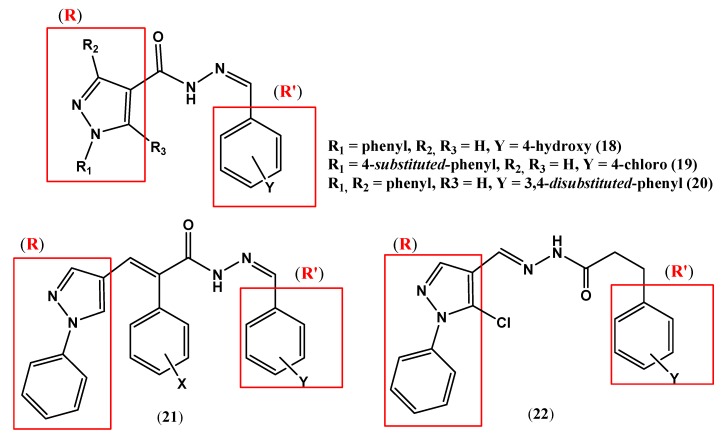
1-*H*-pyrazole-4-carbohydrazide derivatives which show antimicrobial activity (compounds **18**–**21**) and anticonvulsant activity (compound **22**).

The insertion of a spacer unit (ethylenic moiety) between the aromatic subunit (**R**) and carbohydrazide function (Figure 5) increases the distance from the aromatic units (**R** and **R'**) that were previously separated only by the carbohydrazide moiety. A phenyl aromatic ring with different substituents inserted into the ethylenic portion increased the lipophilicity and changed the stereoelectronic factors of these derivatives. These compounds presented trypanocidal activity, especially the compound **21** that was shown to be the most potent and to have the lowest toxicity from this series [33]. The reversal of position of the aromatic units (**R** and **R'**) as in derivative **22** exhibited anticonvulsant effects when evaluated by the standard pentylenetetrazole test and maximum electroshock seizure models [34].

## 4. Conclusions

The biological activity of the various pyrazole derivatives containing carbohydrazide moieties discussed in this paper includes antidepressant, anticonvulsant, analgesic, anti-inflammatory, anticancer, antimicrobial, antinociceptive, and antiparasitic activity, such as, antimalarial, trypanocidal, and leishmanicidal. Even though they have a high significance in the pharmaceutical and biotechnological field with a wide spectrum of biological activities for their various derivatives, one must highlight that the carbohydrazide pyrazole chemical class could possess other biological profiles as they are found in many pharmaceutical lead molecules.
